# Active Protein Aggregates Produced in *Escherichia coli*

**DOI:** 10.3390/ijms12118275

**Published:** 2011-11-22

**Authors:** Špela Peternel, Radovan Komel

**Affiliations:** 1 Laboratory for Biosynthesis and Biotransformation, National Institute of Chemistry, Hajdrihova 19, 1000 Ljubljana, Slovenia; E-Mail: radovan.komel@mf.uni-lj.si; 2 Medical Centre for Molecular Biology, Medical faculty, University of Ljubljana, Vrazov trg 2, 1000 Ljubljana, Slovenia

**Keywords:** *E. coli*, active protein aggregates, inclusion bodies, IBs

## Abstract

Since recombinant proteins are widely used in industry and in research, the need for their low-cost production is increasing. *Escherichia coli* is one of the best known and most often used host organisms for economical protein production. However, upon over-expression, protein aggregates called inclusion bodies (IBs) are often formed. Until recently IBs formation represented a bottleneck in protein production as they were considered as deposits of inactive proteins. However, recent studies show that by choosing the appropriate host strain and designing an optimal production process, IBs composed from properly folded and biologically active recombinant proteins can be prepared. Such active protein particles can be further used for the isolation of pure proteins or as whole active protein particles in various biomedical and other applications. Therefore interest in understanding the mechanisms of their formation as well as their properties is increasing.

## 1. Introduction

The need for low-cost protein production is increasing as the use of proteins is expanding to various areas; from research to a range of commercial applications in pharmaceutical, chemical and food industry, cosmetics as well as biomedical applications (e.g., tissue cultures) and diagnostics. Bacterial host systems for recombinant protein production are still very attractive, as they are usually genetically well-characterized having a large number of cloning vectors and mutant host strains available and they grow rapidly at high density on inexpensive substrates. It is difficult to decide which combination of host organism-promoter system would be the best for specific recombinant protein production; therefore this still has to be optimized for each product individually.

Since *Escherichia coli* have all the above described advantages [1] and the laboratory strains are regarded as GRAS (Generally Recognized As Safe) organisms, it is still one of the most commonly used bacterial host system, not only on laboratory scale use, but also for production of therapeutic proteins [2–5].

However, during recombinant protein production in *E. coli*, proteins often tend to aggregate into protein particles called inclusion bodies (IBs). Until recently, IBs were considered as deposits of misfolded and inactive proteins and represented bottleneck in recombinant protein production. Therefore many pharmaceutically interesting proteins have been disregarded for commercialization [6]. Nevertheless, the latest studies on protein aggregation have shown that protein aggregation into IBs does not necessarily imply protein inactivation [7–10] thus studies on protein aggregation has become an important subject in many fields, including biology, medicine and biotechnology [11]. This article gives an overview on the latest trends in recombinant protein production in *E. coli*, their aggregation and the possible applications of such protein particles (IBs).

## 2. *E. coli* and Recombinant Protein Production

*E. coli* is one of the most widely used hosts for the production of recombinant proteins. Nevertheless, choosing an optimal expression system is vital for an efficient protein production process and is often dependant on recombinant protein itself. There are many different *E. coli* strains and vector systems, however B strains, such as BL21, combined with pET vectors, became popular due to their efficiency in recombinant protein production [2,12]. Because of well controlled promoters, this bacterial factory enables high recombinant protein production yields (up to 50% of total cell proteins). It proved to be a very efficient system also for the production of active protein particles called non-classical IBs (ncIBs) [7,13–15].

However, as recombinant protein production represents stress for the host cell and thus the whole cell machinery has to adapt to an over-expression of foreign protein, quality product can only be produced when the whole bioprocess is optimized [15].

Therefore in addition to the host organism, a production media should also be optimized and quality inoculum prepared for the reproducibility of the production process. Addition of some essential microelements to the basic LB media enables higher accumulation of recombinant proteins [16]. Furthermore, lowering the cultivation temperature was proven to effectively limit the *in vivo* aggregation of recombinant proteins [17], hence higher amounts of soluble proteins are formed [18] and the quality of the proteins is improved, including the ones trapped inside IBs [10,13,14,19,20]. Suboptimal growth temperatures slow down all the cell processes, including transcription and translation [17] thus proteins have more time to fold properly. The extended time period between synthesis and deposition, as well as rapidly exceeded solubility of target protein, results in aggregation of better folded proteins [20,21].

While satisfactory protein yields could be achieved in the shake flask culture, extremely high yields could only be obtained by high cell density fermentation, using finely tuned expression systems [22]. Yet strong production of recombinant proteins and thus unusually high transcription rates could result in a stressful situation for the host cell [23] that has a negative impact on productivity and protein quality [24]. Therefore a fragile balance between high culture density and high protein yield *versus* high protein quality should be maintained for optimal results.

## 3. Protein Aggregation and IBs Formation

The aim of recombinant protein production is to yield high amounts of the desired proteins. As a result the host organism is often forced to produce proteins above the cells physiological capacity. Upon over-expression, a high amount of proteins are constantly formed that cannot be simultaneously processed by the protein synthesis machinery, thus the quality control system is activated [25]. Additionally, *E. coli* has simple protein folding machinery, which lacks post–translational modifications. Due to the reducing potential of the *E. coli* cytoplasmic redox state, the production of proteins possessing more disulfide bonds still remains a challenge. The combination of all these factors, together with the exceeded solubility of over-expressed recombinant protein leads to the aggregation of recombinant proteins, which often gives rise to IBs formation.

IBs were for a long time understood as inert deposits of misfolded and inactive proteins in some way separated from the cellular repair mechanism. However, it is now known that protein aggregation into IBs is reversible [26] and dependent on the physiological state of the host organism. IBs are very dynamic structures that are continuously formed in the host cell and simultaneously the proteins are also released from them and refolded or degraded by the cell repair mechanisms [27].

Since protein aggregates represented an obstacle in recombinant protein production, various mechanisms that would enable production of soluble proteins in bacteria were studied. Yields of soluble protein expression can be increased by chaperone co-expression [28], fusion of target protein with suitable fusion tags [29], choosing appropriate host strain and promoter system or modifying the cultivation conditions (e.g., growth temperature, media composition) [17]. However, studies showed, that protein solubility does not imply protein activity, as large amount of the proteins found soluble in the cytoplasm can be biologically inactive [7,8].

**Figure 1:**
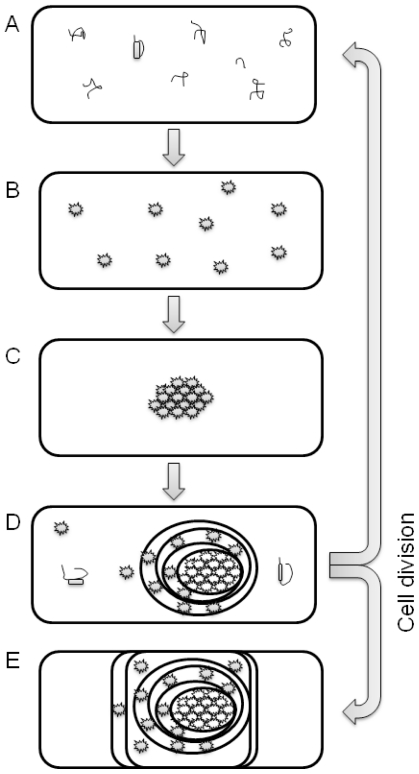
Formation of inclusion bodies (IBs). Since bacterial cytoplasm is not compartmentalized, proteins start to form on multiple locations. During o over-expression of foreign genes, the cell repair mechanisms are overloaded thus various transitional folding states of the recombinant protein are simultaneously present in the cell. Due to various reasons (hydrophobicity, exceeded solubility, cross-molecular stereospecific interactions) these protein precursors start t to aggregate into small “proto-aggregates” t that are then glued together into (usually) a single IB. This IB grows in the cell as a sphere u until it reaches the bacterial cell wall and it is t then prolonged into a cylinder. When the cell divides, the IBs stay in one of the cells and grow further, while the other cell remains e empty and protein production and IB formation begins *de novo*. Studies show that the process of IB translocation to the cell pole before cell division is an energy dependent p process [37].

**Figure 2:**
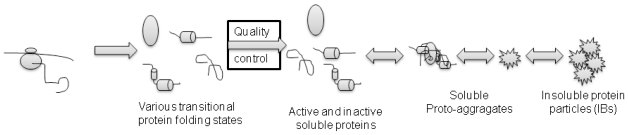
Protein folding and aggregation in *E. coli*. Many proteins fail to o fold to the native conformation during protein n synthesis, therefore various transitional fold ding intermediates are present in the cell together with properly folded proteins. Cells s’ quality control machinery maintains kinetic c equilibrium between soluble and aggregated forms of the protein. Soluble fraction is s composed of single protein molecules as s well as soluble aggregates. Inside the soluble aggregates, properly folded proteins are also trapped. Soluble aggregates are further aggregated into insoluble aggregates called inclusion bodies. The process in reversible and it is s controlled by the cell quality control mechanism.

**Figure 3:**
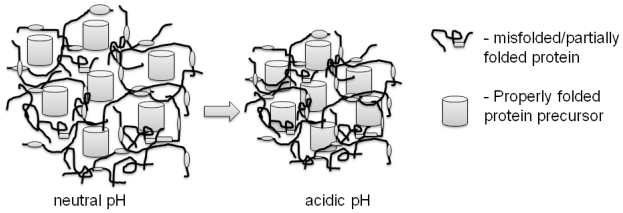
Network of proteins inside IB. IBs are composed from m the network of misfolded/partially folded proteins inside which the properly folded proteins are trapped. The protein network is loosely connected at neutral pH. The transfer of I IBs into acidic pH (around 4) results in strong contraction of the protein network, thus the IBs s are more compact and the properly folded proteins are trapped inside and their extraction is prevented.

The formation of protein aggregates is a self-assembly process in bacterial cells. As there is no compartmentalization, proteins are simultaneously synthesized on multiple locations in the bacterial cytoplasm and various transitional folding states of the target protein are formed (Figure 1). Some of the folding intermediates that fail to fold into a native conformation are immediately degraded by the cells’ repair mechanisms, while others aggregate into smaller proto-aggregates (“soluble aggregates”) [30]. During this nucleation, predominantly target recombinant protein is incorporated into the proto-aggregates by cross-molecular stereospecific interactions, while the other non-homologous cellular (and even recombinant) proteins are excluded from this seeding events [25]. This leads to the rapid growth of proto-aggregates that are later fused together in an IB that continually grows further to form the final IB as depicted in Figure 2 [21,31]. Inside the IB, the network of partially folded proteins is formed and studies show that this network has an amyloid-like structure [32]. Properly folded protein precursors are trapped into this network (Figure 3) [21]. A similar self-assembly process of amyloid-like structures has also been observed in yeasts [33], fungi [34], plants [35] and can be observed in many mammalian (human as well as animal) degenerative disorders formation is an ubiquitous process of many different organisms.

**Figure 4:**
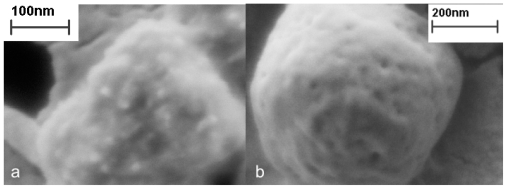
IB observed with a scanning electron microscope. The IBs washed with water (**b**) and additionally rapidly washed in mild detergent (**a**) [7]. It seems that IBs are composed of small proto-aggregates imbedded into a cotton-like amorphous matrix (**a**). However, the amorphous matrix fills the spaces both among and inside the proto-aggregates which gives the IB a porous surface (**b**).

It seems that after the proto-aggregates are fused into a single IB and this IB grows further, the spaces between the proto-aggregates are filled with a mass of proteins incorporated to IB by hydrophobic or stereospecific interactions. If the surface proteins are removed from the IBs’ surface with a mild detergent [7], these p proto-aggregates can be observed under electron microscope and it seems as if they were imbedded into a cotton-like amorphous matrix (Figure 4a). This amorphous matrix fills the spaces both among g and inside the proto-aggregates which gives s the IB a porous surface (Figure 4b).

## 3. Properties of Properly Folded Protein Aggregates

The shape and size of IBs is very much dependant on the host bacterial strain. So the IBs were found to be spherical [7,38], ellipsoidal [31,39], cylindrical [40,41] and even tear-shaped [42] ranging in size form 50–700 nm [7,42].

There is usually one and only rarely two IBs present in the bacterial cell. So after the bacterial cell division, when two cells emerge, IB is present in only one cell while the other cell is empty. Recent study showed that IB is actively translocated to the cell pole before cell division and in this manner aggregated proteins can be removed from bacterial population in nature [37]. In bacterial culture where protein production is artificially induced, production of recombinant proteins in the vacant cells begins *de novo* and a new IB is formed (Figure 1), while the IBs that remain in the cells after cell division grow further. Therefore there are various populations of bacterial cells present simultaneously in the bacterial culture.

**Figure 5:**
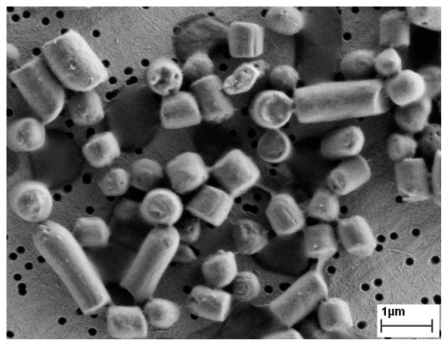
The diverse population of IBs after 24 h production. The protein production starts immediately after the inducer is added to the medium. Therefore some of the IBs in the population have been growing for 24 h and formed cylinders that occupy almost the entire cell space. In contrast, other IBs started growing *de novo* after each bacterial cell division. Therefore the whole range from small spherical IBs, that have been formed in the final hour of cultivation, to large spheres that have been growing for several hours all the way to the cylinders are simultaneously present in the one sample.

Thus the shape and size of IBs is dependant not only on the host strain, but also on the time of cultivation. Based on our observation in the case of spherical IBs, they form spheres at the beginning of cultivation. However, when the IB grows and reaches the bacterial cell wall and it can no longer grow in one direction it starts to form the cylinder. Therefore, the population of IBs after long-term cultivation (24 h) is very diverse as some of the IBs in the population have been growing for 24 h and have formed large cylinders that nearly occupy the whole bacterial cell, ranging from smaller cylinders and large spheres all the way to small spheres that are just beginning to form (Figure 5).

The activity of proteins inside IBs varies significantly, depending on the target protein as well as the production conditions. The active protein particles, ncIBs, are composed from significant amounts of properly folded and biologically active proteins, trapped into the network of misfolded proteins. Thus they possess some interesting properties that can be exploited for various biotechnological, as well as biomedical, applications. Such ncIBs are extremely fragile and soluble in contrast to classical IBs. They are soluble in mild detergents and even in buffers usually used to wash and store classical IBs [7,13]. Therefore optimization of isolation and washing process is necessary in order not to impair the structure of IBs or the protein trapped inside [40,43]. It was shown that sonication, often used for bacterial cell disruption, can damage the structure of ncIBs. Furthermore, the structure of properly folded proteins trapped inside ncIBs can also be destroyed and significant proportion of biologically active protein can thus be lost [40].

Another interesting property of IBs (classical as well as non-classical) is their irreversible contraction at low pH. It seems that the high proton concentration in acidic buffers induces a change in the network of unfolded/partially folded proteins and this leads to a strong contraction of the protein network and formation of more compact IBs [21]. Consequently the solubility of contracted ncIBs is greatly reduced. While at neutral pH the protein network is loosely bound, the extraction of native-like precursor molecules as well as some still soluble proteins having biologically inactive conformations is easy (Figure 3a) [8,13]. However, the contraction of protein network in acidic pH traps the properly folded precursors into the IB and their extraction prevented (Figure 3b). Thus when soluble ncIBs are needed for further applications, the buffers used for washing and storing the IBs should be carefully chosen to prevent ncIBs contraction.

## 4. What Can We Learn from Properly Folded Protein Aggregates and How Can We Use Them?

Understanding protein folding and mechanisms of protein aggregates (IBs) formation in bacteria can serve as a model for protein aggregation in higher organisms and help us understand how and why human conformational diseases progress [32]. Besides this, understanding the properties of IBs could also give us an insight into the properties of protein aggregates formed in mammalian cells. This knowledge could serve as a background for identification of novel target sites for the development of novel and more efficient treatments for human conformational diseases.

Furthermore, IBs, formed after over-expression of recombinant proteins in bacteria, are a highly pure protein deposit of target recombinant protein as it can represent more than 95% of all protein present inside the IBs [44]. Historically, IBs were considered as the main obstacle in protein production while isolating active proteins from IBs represented a great challenge. However, ncIBs composed from significant amount of properly folded and biologically active proteins, easily soluble in mild detergents, are ideal for protein isolation. Since ncIBs are extremely fragile and soluble, bacterial cell disruption process should be optimized. After bacterial cell disruption ncIBs are washed and collected with centrifugation, and the majority of the impurities are removed already in this step. The buffers used during cell disruption and for ncIBs washing should also be carefully chosen, not to dissolve the IBs [7,13,40,43]. While ncIBs are soluble in mild detergents, recombinant protein can be extracted from them in mild non-denaturing conditions in biologically active form, therefore no renaturation step is needed. The protein isolation process is thus simplified, less protein specific, more cost effective and environment-friendlier [7,13–15,45,46]. Since the need for recombinant proteins is constantly increasing, this process could be well exploited in the future.

In the past few years with the development of nanobiotechnology the field of possible applications of protein particles (IBs) is broadening and protein micro- and nano-particles are becoming increasingly interesting. The IBs are therefore studied for various biomedical applications [47].

As IBs are large protein particles that can easily trigger the immune response, the idea of using the whole IBs as a vaccine was tested by several different research groups simultaneously. Successful protective immunity was reported for vaccines against several animal diseases (e.g., liver fluke, classical swine fever, salmonid rikettsial septicaemia …) [48–50] as well as against human oral infections (gingivitis and periodontitis) [51].

Furthermore, IBs composed from a suitable protein (pseudan) can be used as a coating in medical tubes to reduce or even inhibit biofilm formation [52]. On the other hand, recent studies showed, that IBs can be utilized as a scaffold material in tissue engineering, as they affect the mammalian cell attachment and proliferation [39,42,53].

However, the protein particles employed in these applications were described as classical, insoluble IBs and the activity (proper folding) of the entrapped proteins is not discussed. With the preparation of active protein particles, the scope of possible applications is even broader. As a result Nahálka and co-workers prepared IBs composed from a wide variety of different biologically active enzymes that were shown to act as biocatalysts [54–59] and could also be used for the development of new diagnostic techniques [60]. In addition, soluble ncIBs could also be used as protein delivery system.

Furthermore, coating of IBs with various coatings was shown to additionally stabilize IBs structure and even enhance enzymatic activity of these active protein particles [58,59].

The field of nanobiotechnology is new and fast evolving, thus predicting various possibilities of IBs applications is difficult. However, it could be anticipated that such nanoparticles could be used in the development of new treatments and diagnostic techniques in medicine, biomedicine and pharmacology as well as biocatalysts in various industries (cosmetics, biotechnology, food and chemical industry) [47,61,62].

## 5. Conclusion and Perspectives

Protein aggregation into IBs has long been considered as an obstacle in protein production in bacteria. However, the knowledge gained during studies of protein aggregation in bacteria could help us understand the human progressive conformational diseases that represent a growing problem with increased prevalence in an aging society.

Furthermore, with carefully designed bioprocess production of active and even soluble protein aggregates being possible, such aggregates can be used in biotechnology for the isolation of pure recombinant proteins and as micro-/nanoparticles in various biomedical and pharmacological applications as well as in other fields.

As this is still an emerging and fast evolving discipline, the protocols for production and preparation of protein particles have to be redesigned and carefully optimized. A more thorough and systematic study on production and properties of such protein particles is needed in order to enable the design of particles with the desired properties (e.g., solubility, biological activity, size, shape…) [62].
